# Efficacy of etoricoxib for ankylosing spondylitis: A protocol for systematic review of randomized controlled trial

**DOI:** 10.1097/MD.0000000000015155

**Published:** 2019-04-12

**Authors:** Hua-yu Tang, Yu Zhao, Yu-zhi Li, Tian-shu Wang

**Affiliations:** a Second Ward of Orthopedis Department, First Affiliated Hospital of Jiamusi University, Jiamusi, China; b Department of Orthopedis, Huludao Central Hospital, Huludao, China; c Department of Urology, First Affiliated Hospital of Jiamusi University, Jiamusi, China.

**Keywords:** ankylosing spondylitis, efficacy, etoricoxib, safety

## Abstract

**Background:**

Previous clinical trials have reported that etoricoxib has been utilized to treat ankylosing spondylitis (AS) effectively. However, no study systematically investigated the efficacy and safety of etoricoxib for patients with AS. In this systematic review, we will assess the efficacy and safety of etoricoxib for AS.

**Methods:**

The following electronic databases will be searched from inception to the February 1, 2019: Cochrane Library, Embase, PubMed, Cumulative Index to Nursing and Allied Health Literature, China National Knowledge Infrastructure, Chinese Biomedical Literature Database, and Chinese Scientific Journal Database. This study will include randomized controlled trials that explore the efficacy and safety of etoricoxib for AS. The primary outcome is pain intensity, as measured by any pain scales, such as Numerical Rating Scale. The secondary outcomes consist of AS function, as measured by Bath Ankylosing Spondylitis Functional Index, or other tools; quality of life, as assessed by Ankylosing Spondylitis Quality of Life questionnaire or any other relevant instruments; as well as adverse events. Two authors will independently carry out the study selection, data extraction, and risk of bias assessment. Statistical analysis will be performed by using RevMan 5.3 software.

**Results:**

This systematic review will provide a detailed summary of present evidence related to the efficacy and safety of etoricoxib for patients with AS.

**Conclusion:**

The results of this study may provide management guidance for AS treated by etoricoxib.

**Dissemination and ethics:**

This systematic review dose not needs ethical approval, because it will not analyze individual patient data. The findings of this study are expected to publish through a peer-reviewed journal.

**Systematic review registration:**

CRD42019124768.

## 1 Introduction

Ankylosing spondylitis (AS) is a common chronic rheumatic disorder.^[1–3]^ Patients experience such condition often complain with pain in the area of ankle, eyes, heel, hip, joints, lower back, middle back, neck, or shoulder; blurred vision; and back joint dysfunction or stiffness.^[4–7]^ It is estimated that its prevalence varies from the 0.1 to 1.4%,^[8]^ with male-to-female ratio ranges from 2:1 to 3:1.^[9]^ If it cannot be treated effectively, it finally leads to very poor quality of life.

A variety of clinical trials have reported that etoricoxib can be used to treated AS, and have achieved very exciting outcome results.^[10–21]^ However, no systematic review and meta-analysis has been conducted to assess the efficacy and safety of etoricoxib for the treatment of patients with AS. Therefore, in this study, we systematically evaluate the efficacy and safety of etoricoxib for patient with AS.

## 2 Methods and analysis

### 2.1 Eligibility criteria for study selection

#### 2.1.1 Types of study

Randomized controlled trials (RCTs) investigating the efficacy and safety of etoricoxib for AS will be included. However, the studies belong to the nonclinical trials, case studies, noncontrol studies, and non-RCTs will all be excluded.

#### 2.1.2 Types of participant

Participants with clinically diagnosed of AS will be fully considered for inclusion regardless the race, age, and gender.

#### 2.1.3 Types of intervention

The experimental group utilized etoricoxib monotherapy. The control group can use any interventions, except any forms of etoricoxib.

#### 2.1.4 Types of outcome measurement

The primary outcome of pain intensity can be measured by any pain scales, such as Numerical Rating Scale.

The secondary outcomes comprise of AS function, as assessed by Bath Ankylosing Spondylitis Functional Index any associated tools; health-related quality of life, as evaluated by any instruments, such as Ankylosing Spondylitis Quality of Life questionnaire; as well as adverse events.

### 2.2 Literature search

We will search the electronic databases of Cochrane Library, Embase, PubMed, Cumulative Index to Nursing and Allied Health Literature, China National Knowledge Infrastructure, Chinese Biomedical Literature Database, and Chinese Scientific Journal Database from all their inceptions to the February 1, 2019 without any language restrictions. Furthermore, reference lists of all eligible trials and relevant reviews will be searched. The detailed search strategy for Cochrane Library is shown in Table 1. Additionally, similar search strategy details are also applied to any other electronic databases.

**Table 1:**
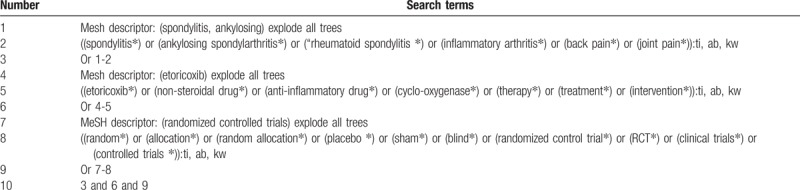
Search strategy utilized for Cochrane Library database.

### 2.3 Study selection

Two authors will independently scan the title, abstract for each potential study initially in accordance with the predefined eligibility criteria. Then, full texts will be read to further judge if those studies are eligible or not for final inclusion after the initial selection. Any disagreements regarding the study selection between 2 authors will be solved by a third author through discussion. All process of study selection abides to the Preferred Reporting Items for Systematic Reviews and Meta-Analyses (PRISMA)^[22]^ and PRISMA-Protocol guidelines.^[23,24]^ Moreover, a PRISMA flowchart will be applied to study selection with details reasons of exclusion and inclusion for each study at each phrase.

### 2.4 Data extraction and management

All data will be independently extracted and collected by 2 authors using a predefined standardized data extraction sheet. The extracted information includes title, authors, year of publication, country; patient characteristics, such as age, race, sex, diagnostic criteria, inclusion and exclusion criteria; study method, such as sample size, randomization, concealment, blinding, and any other potential risk of bias; intervention details, such as dosage, duration, frequency; and outcome measurements, such as primary, secondary, and any other outcome tools.

### 2.5 Missing data dealing with

If any insufficient or missing data is occurred, we will contact the primary corresponding author to inquire those data. If those data cannot be achieved, then just the present data will be analyzed.

### 2.6 Risk of bias assessment

Risk of bias for each included study will be judged by using Cochrane Risk of Bias Tool. Two authors will independently judge them for each study on 7 aspects. Each aspect will be categorized as high, unclear or low risk of bias. Any disagreements regarding the risk of bias assessment will be solved by a third author through discussion.

### 2.7 Statistical analysis

RevMan 5.3 software will be used for statistical analysis, including pooling data, and meta-analysis conduction.

#### 2.7.1 Treatment effect measurement

We will describe treatment effects using risk ratio and 95% confidence intervals (CIs) for dichotomous outcome values; and mean difference, or standardized mean difference and 95% CIs for continuous outcome values.

#### 2.7.2 Heterogeneity evaluation

Heterogeneity will be evaluated by *I*^*2*^ test. If *I*^*2*^ ≤ 50%, minor heterogeneity will be regarded. On the other hand, if *I*^*2*^ > 50%, substantial heterogeneity will be considered in this study.

#### 2.7.3 Data synthesis

If heterogeneity is minor, data will be pooled, and a fixed-effect model will be used to pool the data. In addition, meta-analysis will be carried out. On the other hand, if heterogeneity is substantial, a random-effect model will be utilized to pool the data, and subgroup analysis will be performed. If there is minor heterogeneity after the subgroup analysis, meta-analysis will be conducted. Otherwise, if there is still significant heterogeneity after the subgroup analysis, meta-analysis will not be operated. Instead, a narrative summary will be reported.

#### 2.7.4 Subgroup analysis

We will carry out subgroup analysis in accordance with the different characteristics, treatments, controls, and outcome instruments.

#### 2.7.5 Sensitivity analysis

We will perform the sensitivity analysis to examine the robustness of the pooled outcomes by removing low quality of trials.

#### 2.7.6 Publication bias

We will operate the funnel plot and Egger's test to detect any publication bias if more than 10 RCTs are included in this study.

## 3 Discussion

This study firstly assessed the efficacy and safety of etoricoxib for patients with AS. The results will provide a detailed summary of the current evidence relevant of etoricoxib in pain relief, AS function improvement, and health related quality of life enhancement in patients with AS. Moreover, its findings may also provide helpful evidence for both clinician and patients.

## Author contributions

**Conceptualization:** Hua-yu Tang, Yu Zhao, Tian-shu Wang.

**Data curation:** Hua-yu Tang, Yu-zhi Li, Tian-shu Wang.

**Formal analysis:** Hua-yu Tang, Yu Zhao, Yu-zhi Li.

**Funding acquisition:** Hua-yu Tang.

**Investigation:** Tian-shu Wang.

**Methodology:** Hua-yu Tang, Yu Zhao, Yu-zhi Li.

**Project administration:** Tian-shu Wang.

**Resources:** Hua-yu Tang, Yu Zhao, Yu-zhi Li.

**Software:** Hua-yu Tang, Yu Zhao, Yu-zhi Li.

**Supervision:** Tian-shu Wang.

**Validation:** Yu-zhi Li, Tian-shu Wang.

**Visualization:** Hua-yu Tang, Yu Zhao, Yu-zhi Li, Tian-shu Wang.

**Writing – original draft:** Hua-yu Tang, Yu Zhao, Yu-zhi Li, Tian-shu Wang.

**Writing – review & editing:** Hua-yu Tang, Yu Zhao, Yu-zhi Li, Tian-shu Wang.
